# Autoantibodies to speckled protein family in primary biliary cholangitis 

**DOI:** 10.1186/s13223-021-00539-0

**Published:** 2021-03-31

**Authors:** Alessandro Granito, Luigi Muratori, Francesco Tovoli, Paolo Muratori

**Affiliations:** 1 Division of Internal Medicine, IRCCS Azienda Ospedaliero-Universitaria di Bologna, 40138 Bologna, Italy; 2 Center for the Study, Treatment of Autoimmune Diseases of the Liver, Biliary System, Bologna, Italy; 3 Department of Medical and Surgical Sciences (DIMEC), Alma Mater Studiorum, University of Bologna, Bologna, Italy; 4 Department for the Science of the Quality of Life (QUVI), Alma Mater Studiorum, University of Bologna, Bologna, Italy

**Keywords:** Speckled proteins, Multiple nuclear dots, Antinuclear antibodies, Primary biliary cholangitis

## Abstract

The autoantibody profile of primary biliary cholangitis (PBC) includes antinuclear antibodies (ANA) which are detectable by indirect immunofluorescence in more than 50% of PBC patients. One of the two immunofluorescence patterns which are historically considered “PBC-specific” is the so-called “multiple nuclear dots” (MND) targeting nuclear body proteins such as Sp100, Sp140, Sp140L proteins, promyelocytic leukemia protein (PML) and small ubiquitin-related modifier proteins (SUMO). It has been hypothesized a role of nuclear body protein alterations in immune disorders such as PBC, thus suggesting novel and more refined therapeutic approaches.

To the editor

Patients with primary biliary cholangitis (PBC) produce antinuclear antibodies (ANA) directed against structural components of promyelocytic leukemia protein (PML) and Sp100-containing nuclear bodies (NBs) [1].

Sp100, PML, Sp140, Sp140L, and small ubiquitin-related modifier (SUMO) proteins are PML NB-related proteins that are identified as target antigens in PBC patients [2–6]. These autoantibodies are of clinical relevance in PBC due to their very high disease specificity and as surrogate markers in anti-mitochondrial antibody (AMA) negative PBC cases (Table 1) [1, 2].

**Table 1:** Relationship between nuclear pore complex proteins, antinuclear antibodies giving the “multiple nuclear dots” immunofluorescence pattern, and clinical significance in patients with primary biliary cholangitis

Nuclear structures	Indirect immunofluorescence pattern	Autoantigen targets	Autoantibodies in PBC		
			Prevalence (%)	Specificity	Worse prognosis
Nuclear Bodies	MND^a^	Sp100	17–41	High	To be confirmed
		PML	19	High	No
		Sp140	15	High	No
		Sp140L	1.5	Not assessed	Not assessed
		SUMO-1	15	Not assessed	Not assessed
		SUMO-2	42	Not assessed	Not assessed

*Sp100* Sp100 nuclear body component, *PML* promyelocytic leukemia protein, *Sp140* Sp140 nuclear body component, *Sp140L* Sp140L protein, *MND* multiple nuclear dots pattern, *SUMO* small ubiquitin-related modifiers

^a^MND pattern detected by indirect immunofluorescence using fluorescein-conjugate anti-human total Ig and anti-human IgG subclasses (IgG1, IgG2, IgG3, IgG4) as secondary antibody was found in 16% and 31%, respectively [1, 2]

Within the spectrum of ANA staining patterns by indirect immunofluorescence, the “multiple nuclear dots” (MND) pattern is therefore historically considered as highly specific for PBC [1, 7].

The MND staining pattern is characterized by the presence of 5–20 dots of variable size distributed throughout the cell nucleus but sparing the nucleoli; it is distinguishable from the centromere staining pattern by the presence of fewer nuclear dots per cell, and by the absence of dots in cells that are undergoing mitosis (Fig. 1) [7]. The MND staining pattern is also distinct from the anti-p80 coilin/Cajal body staining pattern, which is characterized by the presence of 2–8 dots per cell nucleus [8].

**Fig. 1:**
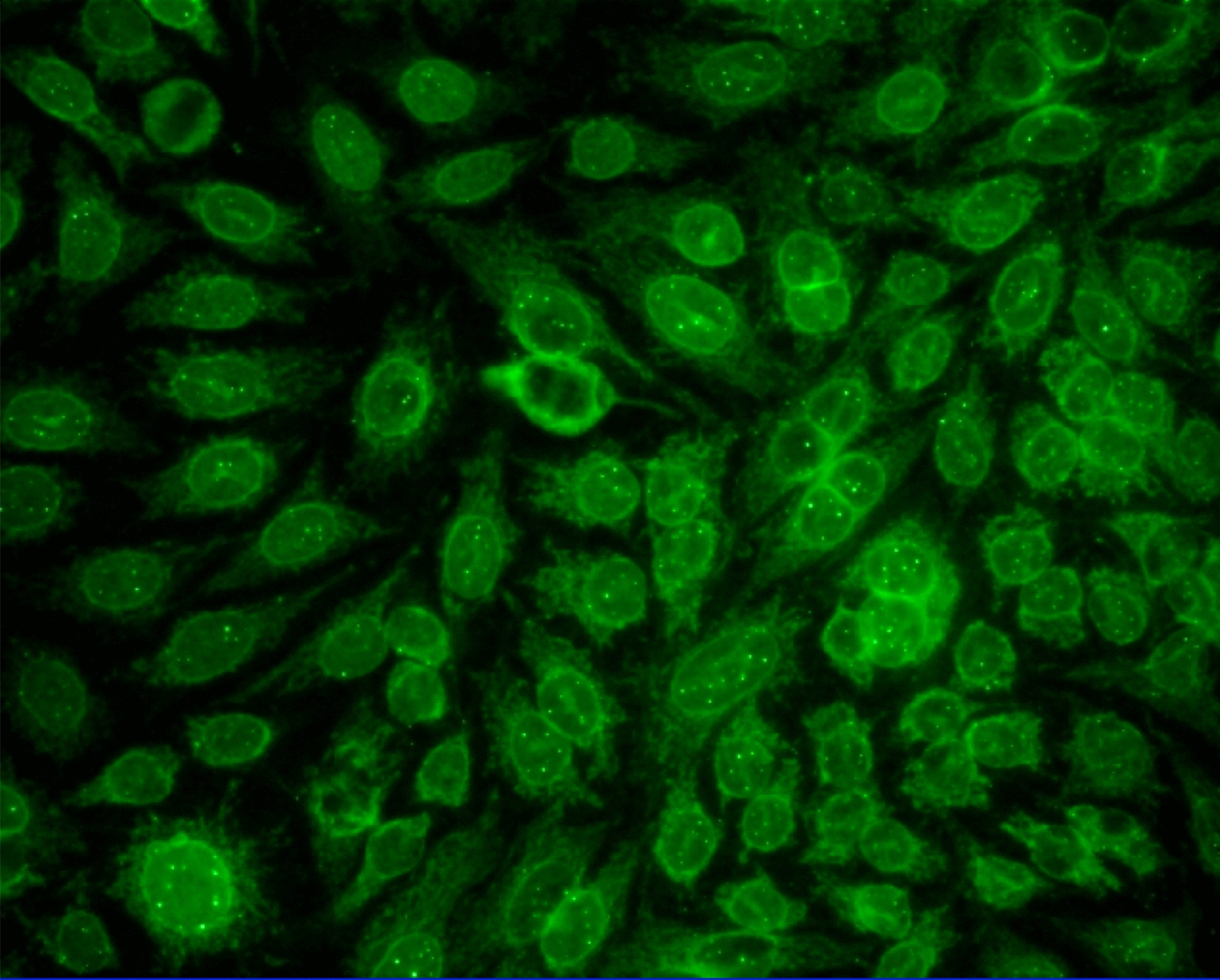
Multiple nuclear dot staining pattern by indirect immunofluorescence on HEp-2 cells (magnification 20). Anti-multiple nuclear dots react with 3–20 nuclear dots distinct from nucleoli and from the anticentromere targets. Punctate staining of chromosomes in mitosis clearly distinguishes anticentromere from anti-multiple nuclear dots

The mechanism leading to ANA production in PBC is still an unsolved question. It has been suggested that xenobiotics and molecular mimicry between microbial agents and self-antigens might be involved in the triggering of disease as well as in the appearance of autoantibodies [9, 10].

Previous data suggest that PML-NBs may have a role in transcriptional events [11]. Moreover, it has been shown that PML, Sp100, and Sp140 are upregulated in response to interferons, a group of proteins with antiviral activities, indicating that PML NBs could also have an important function in antiviral defense [12]. Results from a recent study suggest an implication of Sp140 protein in an innate response to HIV-1 by its interaction with the *vif* protein encoded by the virus [13].

In their review article, Fraschilla and Jeffrey posit that the speckled protein (SP) family are central chromatin regulators of gene silencing that establish immune cell identity and function [14]. They correctly point out that: (1) mutations in human SP140 associate with three immunological diseases: Crohn’s disease, chronic lymphocytic leukemia, and multiple sclerosis; (2) mutations in human SP110 associate with veno-occlusive disease with immunodeficiency; (3) finally, many viruses have evolved mechanisms to inhibit SP function in PML-NBs, organelles associated with viral gene repression, suggesting that SPs mediate protective viral defense mechanisms [15].

They conclude that all SPs are associated with autoimmune, inflammatory, or infectious diseases, underscoring their role in maintaining immune homeostasis and proper functional response to pathogens.

Regarding their role in PBC, it has been widely established the high diagnostic value of autoantibodies direct against SPs, especially in patients lacking antimitochondrial antibodies. Moreover, a prognostic role for MND/anti-Sp100 antibodies has also been suggested: Zuchner et al. described a faster disease progression among anti-Sp100-positive patients with PBC [16]. Rigopoulou et al. reported that anti-MND-positive patients had significantly more severe liver disease than those that were anti-MND negative, as shown by the higher frequency of cirrhosis and worse outcome [17]. However, these observations still need to be confirmed in larger series of patients, possibly recruited from different centers and with different ethnic and genetic backgrounds.

We would like also to add further relevant and unmentioned evidence that supports a potential role of infections as a potential trigger of PBC and anti-SPs autoantibodies development in genetically predisposed individuals. Specifically, it has been demonstrated a possible role of microorganisms that are responsible for recurrent urinary tract infections, as triggers of PBC and ANAs generation, has long been suggested [16–19]. Moreover, a possible molecular mimicry between the epitopic regions of *Escherichia coli* and Sp100 has been hypothesized on the basis of a strong correlation between the presence of anti-Sp100 antibodies and AMA positivity in women with recurrent urinary tract infections, with or without evidence of PBC [20].

A different mimicry mechanism has been proposed by Shinoda et al. who have found that peptides from gp210 and Sp100 proteins are recognized by T-cell clones responsive to the major autoepitope of E2 subunit of the pyruvate dehydrogenase complex. The investigators thus hypothesized that the PBC-specific antinuclear reactivities could be the result of intermolecular spreading involving mitochondrial antigens and mimicry sequences of nuclear proteins [21].

It is noteworthy that more than 80% of anti-NB positive patients has two or three simultaneous anti-NB reactivities, suggesting a “clustering” of autoantigens, an observation supporting the hypothesis that intermolecular epitope-spreading mechanisms might be operative in the propagation of several reactivities in the same patient [4, 22–25].

Given the observation that anti-Sp140, as well as anti-PML antibodies, almost exclusively occur in anti-Sp100-positive patients, but not vice versa, it could be hypothesized that the NB is a multiantigenic complex in which the immune response might involve first Sp100, and only later spread to Sp140 and PML that share the same subnuclear localization.

We agree with Fraschilla and Jeffrey that further studies elucidating mechanisms of SPs alteration, contributing to immune disorders, will aid in the design of new therapies associated with SPs role and function.

Furthermore, immune-expressed SPs may offer novel and more refined therapeutic avenues for taming hyperactive autoimmune responses.

## Data Availability

Data included in this manuscript are available in the following published manuscripts: 1. Muratori P et al. Autoimmunity. 2009;42:224–7. 2. Granito A et al. Am J Gastroenterol 2010;105:125–31. 3. Granito A et al. Aliment Pharmacol Ther. 2006;24:1575–83.

## Abbreviations

PBC: Primary biliary cholangitis
MND: Multiple nuclear dots
NBs: Nuclear bodies
PML: Promyelocytic leukemia protein
SP: Speckled protein
SUMO: Small ubiquitin-related modifier proteins
